# Mapping individual aspects of bilingual experience to adaptations in brain structure

**DOI:** 10.1093/cercor/bhae029

**Published:** 2024-02-13

**Authors:** Vincent DeLuca, Toms Voits, Jianzhang Ni, Felix Carter, Foyzul Rahman, Ali Mazaheri, Andrea Krott, Katrien Segaert

**Affiliations:** Department of Language and Culture, UiT The Arctic University of Norway, Tromso 9019, Norway; Department of Language and Culture, UiT The Arctic University of Norway, Tromso 9019, Norway; Department of Psychology, University of Gothenburg, Gothenburg 405 30, Sweden; School of Psychology and Centre for Human Brain Health, University of Birmingham, Birmingham, B15 2SA, United Kingdom; Department of Psychiatry, The Chinese University of Hong Kong, Sha Tin, Hong Kong; School of Psychological Science, University of Bristol, Bristol, BS8 1TU, United Kingdom; School of Psychology and Centre for Human Brain Health, University of Birmingham, Birmingham, B15 2SA, United Kingdom; College of Psychology, Birmingham City University, Birmingham, B4 7BD, United Kingdom; School of Psychology and Centre for Human Brain Health, University of Birmingham, Birmingham, B15 2SA, United Kingdom; School of Psychology and Centre for Human Brain Health, University of Birmingham, Birmingham, B15 2SA, United Kingdom; School of Psychology and Centre for Human Brain Health, University of Birmingham, Birmingham, B15 2SA, United Kingdom

**Keywords:** bilingualism, individual differences, MRI, neuroplasticity

## Abstract

Individual differences in using multiple languages are thought to differentially affect brain structure and function. The present study assessed the neuroanatomical predictions of an emerging theory, the Unifying the Bilingual Experience Trajectories framework, which provides the most comprehensive set of predictions of how individual differences in bilingual experiences lead to specific neural and cognitive adaptations. A total of 140 young adults with variable language experiences were scanned using magnetic resonance imaging and completed demographic questionnaires. Brain structure measures implicated in predictions of the Unifying the Bilingual Experience Trajectories model were extracted and regressed against the model’s experiential factors. Consistent with the model’s predictions, greater intensity and diversity of bilingual language use resulted in changes in gray matter volume in cortical regions involved in executive control (including inferior frontal gyrus, middle temporal gyrus, angular gyrus, and medial frontal gyrus), indicating adaptations toward handling increased executive control demands. Conversely, duration of bilingual engagement resulted in changes within white matter microstructure (bilateral superior longitudinal fasciculus) and increases in subcortical gray matter (left caudate), indicative of adaptations toward increased efficiency of control. Overall, this research enhances our understanding of how bilingual experiences influence brain structure and provides the first direct empirical evidence for the predictions made by the Unifying the Bilingual Experience Trajectories framework.

## Introduction

A substantial body of research over the past decades has shown that the brain is highly plastic and adaptive to its environment and the cognitive demands associated with it (Fuchs and Flügge 2014). Bilingual language experience is no exception to this. Bilingualism and, crucially, individual differences in the nature and degree of that experience are associated with neuroanatomical adaptations toward maximal effectiveness and/or efficiency at handling the cognitive demands associated with managing multiple languages (Li et al. 2014; Pliatsikas 2019). Recently, theoretical models have proposed specific neurocognitive adaptations to disparate bilingual experiences, including one of the most comprehensive of the models to date, the Unifying the Bilingual Experience Trajectories (UBET) framework (DeLuca et al. 2020). However, as we will unpack below, no study to date has directly examined the neuroanatomical predictions of UBET. We aim to provide empirical evidence and further contribute to the understanding of how different facets of bilingual experience lead to neuroanatomical adaptations which can subserve neurocognitive outcomes, in line with the principles proposed by UBET.

## Background

The mechanisms by which bilingualism induces neurocognitive adaptation center around how the brain manages the languages one speaks. These languages are argued to be constantly and jointly active, creating a state of competition (Green 1998; Marian and Spivey 2003). To facilitate successful communication, this competition is resolved via actively selecting the appropriate language or inhibiting the unneeded language (Abutalebi and Green 2007; Kroll et al. 2012). These processes are cognitively demanding; thus, the brain adapts both structurally and functionally to more effectively handle them (Li et al. 2014; Pliatsikas 2019).

However, bilingualism is not a monolithic experience but a spectrum of distinct and overlapping experiences (Luk and Bialystok 2013; Bak 2016; de Bruin 2019; Titone and Tiv 2023). Given the mechanisms for adaptation to language control demands, differences in language experience should incur varying cognitive demands and, by extension, trajectories of adaptation. A growing body of evidence indicates this: Different language experiences incur distinct neural adaptations in brain regions and networks implicated in cognitive control (e.g. Berken et al. 2016; Kuhl et al. 2016; Nichols and Joanisse 2016; Pliatsikas et al. 2017; Rossi et al. 2017; Luo et al. 2019; DeLuca et al. 2019a; Sulpizio et al. 2020; Fedeli et al. 2021; Marín-Marín et al. 2022). Furthermore, similar bilingual experiences more consistently relate to similar neural adaptations. Neuroanatomically, early and more intensive engagement with multiple languages correlates to increases in gray matter volume (GMV) in cortical regions (e.g. anterior cingulate cortex (ACC), inferior frontal gyrus (IFG), inferior parietal lobule (IPL)) and the hippocampus (e.g. Mårtensson et al. 2012; Bellander et al. 2016; Liu et al. 2021; Marín-Marín et al. 2022). Conversely, prolonged exposure to, or engagement with, multiple languages corresponds to adaptations in white matter structure in tracts connecting language control regions (e.g. corpus callosum (CC), inferior fronto-occipital fasciculus (IFOF), superior longitudinal fasciculus (SLF)) and also within subcortical (e.g. caudate nucleus, thalamus, putamen) and cerebellar gray matter (e.g. Kuhl et al. 2016; Nichols and Joanisse 2016; Pliatsikas et al. 2017; Rossi et al. 2017; DeLuca et al. 2019a; DeLuca et al. 2019b; Korenar et al. 2023). Crucially, the degree to which these adaptations occur is calibrated to the degree of each specific bilingual experience.

### Models of bilingual individual differences

While this initial step is promising in better understanding the nature of bilingualism-induced neural adaptations, calls have been made to employ more theoretically-driven research regarding specific adaptations to bilingual experiences (Blanco-Elorrieta and Caramazza 2021; de Bruin et al. 2021; Leivada et al. 2022). Based on data from both group and individual difference studies in the bilingualism and neurocognition literature, several models have proposed specific neural adaptations to individual differences in bilingual experience. These include the Adaptive Control Hypothesis (ACH; Green and Abutalebi 2013; Abutalebi and Green 2016), the Bilingual Anterior to Posterior and Subcortical Shift framework (BAPSS; Grundy et al. 2017), the Dynamic Restructuring Model (DRM; Pliatsikas 2020), the Conditional routing model (CRM; Stocco et al. 2014), and most recently the UBET framework (DeLuca et al. 2020). Importantly, they make separate, but overlapping, predictions regarding trajectories of neural adaptation to specific bilingual experiences. UBET makes the most comprehensive predictions regarding the mappings of different bilingual experiences to neurocognitive adaptations. As some of the building blocks of UBET were derived from these other models, we first describe other models and then we describe UBET in more detail.

The ACH proposes that more intensive engagement with specific conversational contexts corresponds to reliance on subsections of the language control network (Green and Abutalebi 2013; Abutalebi and Green 2016). Three contexts are proposed: single, dual, and dense code-switching contexts. Single language contexts refer to environments where only one language is used with interlocutors. This context requires the inhibition of the other language and primarily puts demands on the left prefrontal cortex (Calabria et al. 2018). In dual language contexts, both languages are used but with different interlocutors. Such contexts place demands on a range of cognitive processes (inhibition, monitoring, planning, etc.) and a range of cortical and subcortical regions to handle them (including the IFG, IPL, caudate/putamen, ACC, cerebellum). Finally, in dense code-switching contexts, both languages are used with the same interlocutor, often intermixed within the same sentence. Such contexts require predominantly opportunistic planning and rely on a connection between the left prefrontal cortex and the cerebellum. Neuroanatomically, increased engagement with one of these contexts incurs reliance on related aspects of the language control network, thus reinforcing them structurally.

Other models make predictions around the durative aspect of bilingual experience (Stocco et al. 2014; Grundy et al. 2017; Pliatsikas 2020). Neurocognitively, a longer duration of bilingual language engagement is argued to cause a shift in reliance from fronto-cortical regions involved in executive control (EC) toward subcortical and posterior regions as well as the white matter tracts that connect them. Such adaptations reflect a shift in neural organization away from more controlled and toward more automatic processes, achieving maximal efficiency in handling the cognitive demands associated with language control. Of these models, the DRM (Pliatsikas 2020) makes specific predictions for neuroanatomical adaptations. It specifies 3 stages. The first covers early bilingual experience (initial exposure) where the brain must adapt to newly increased control demands and proposes primarily adaptations (i.e. increases) in cortical gray matter (e.g. ACC, IFG IPL, middle frontal gyrus, hippocampus). The second stage (i.e. consolidation) involves adaptations shifts toward efficiency and is characterized by reductions in cortical gray matter (same structures) and increases in white matter microstructure (e.g. IFOF, SLF, CC) as well as subcortical (e.g. putamen, thalamus) and cerebellar gray matter. The final stage (i.e. peak efficiency) is largely proposed as a continuation of the consolidation phase toward maximizing efficiency of language control, which is characterized by reductions in frontal white matter and subcortical gray matter and possible continued increases in cerebellar gray matter. It should be noted that the other 2 models, BAPSS and CRM, converge with the DRM from a mechanistic standpoint: Prolonged L2 use in both models is argued to increase reliance on the basal ganglia and/or other subcortical structures as a measure of increased efficiency of handling processing demands (Stocco et al. 2014; Grundy et al. 2017). Furthermore, the models also overlap in terms of the brain regions implicated in these adaptation trajectories, notably cortical gray matter (e.g. IFG, IPL), and subcortical (basal ganglia)/cerebellar gray matter within the language control network.

**Fig. 1:**
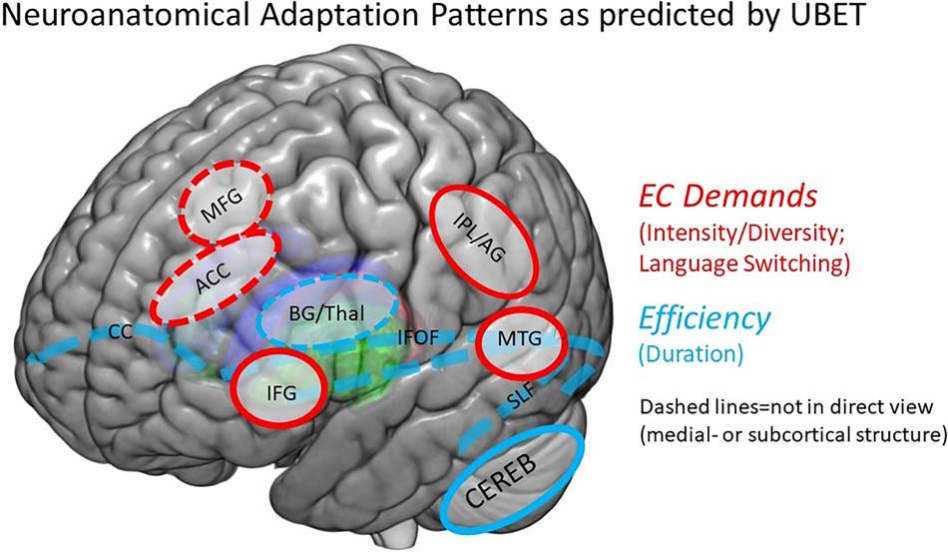
The neuroanatomical predictions of the UBET framework. Red shapes indicate regions implicated in adaptations to increased EC demands (corresponding to degree of engagement in bilingual experience and language switching). Blue lines indicate regions/tracts implicated in adaptations toward efficiency (corresponding to duration of experience). ROI abbreviations: ACC = anterior cingulate cortex, IFG = inferior frontal gyrus, IPL/AG = inferior parietal lobule/angular gyrus, MFG = medial frontal gyrus, MTG = medial temporal gyrus, BG/thal = basal ganglia (caudate/putamen)/thalamus, Cereb = cerebellum, CC = corpus callosum, IFOF = inferior fronto-occipital fasciculus, SLF = superior longitudinal fasciculus.

The UBET framework (DeLuca et al. 2020) has attempted to consolidate the existing theoretical predictions and empirical findings in a more comprehensive, unified model (see Fig. 1 for a schematic representation). UBET proposes a set of bilingual experience-based factors and their specific neural and cognitive effects. Increased diversity and intensity of bilingual use and frequent, controlled language switching is predicted to necessitate adaptations toward increased EC demands. Neuroanatomically, these adaptations should manifest as increases in GMV across cortical regions implicated in EC, such as the ACC, IFG, IPL, medial frontal gyrus (MFG), and middle temporal gyrus (MTG). Conversely, prolonged duration of bilingual experience (and related to that, more balanced fluency between languages) is predicted to lead to adaptations toward increased efficiency in EC. Structurally, the latter adaptations are proposed to manifest as modulations in gray matter structure within the cerebellum and several subcortical structures implicated in EC (including the caudate nucleus, thalamus, and putamen), and higher white matter integrity across tracts that connect different fronto-cortical, parietal, posterior, and subcortical regions, including the CC, IFOF, and SLF). However, to date, the neuroanatomical predictions of the UBET framework have not been directly tested.

### Present study

Herein, we assessed the degree to which the bilingual experience factors proposed within UBET (duration of experience, language switching, intensity/diversity of bilingual use) would predict patterns of neuroanatomical adaptation. Note that we did not assess a further factor specified by UBET, relative proficiency, as this factor shared too much variance with the intensity/diversity of bilingual use factor in our sample (Carter et al. 2023). Moreover, we assessed the extent to which the relationships between (bilingual) experiences and adaptations manifest in a dynamic, nonlinear fashion (Pliatsikas 2020; Marín-Marín et al. 2022; Korenar et al. 2023). We recruited young adult participants with varying degrees of bilingual language experience (including monolingual participants) and took detailed measurements of their language demographics. We also measured their gray and white matter structures via magnetic resonance imaging (MRI). Following UBET, we predicted that increased intensity and diversity of language use and frequent language switching would correspond with increased GMV in cortical regions including the ACC, IFG, IPL, MFG, and MTG. Prolonged duration of bilingual experience would correspond to increases in white matter integrity in tracts including the CC, IFOF, and SLF as well as adaptations in several subcortical structures involved in bilingual language control, including the caudate, thalamus, and putamen.

## Materials and methods

### Participants

The data reported here were collected as part of a larger project combining language demographic/background, cognitive, electroencephalographic (EEG), and MRI measures. For this wider project, 239 participants (163 female, mean age: 22.9 yr, SD: 3.7) were recruited (see Carter et al. 2023) who completed all or some components of the wider project. For the present study, we used only the data of those participants who completed both the MRI and language/demographic (questionnaire) measures (*n* = 155). Of this, data from 15 participants were excluded from the final gray matter analysis; reasons included missing scans (*n* = 2), missing demographic data (*n* = 11), an outlier within the demographic data (*n* = 1), and incidental finding in the structural scan (*n* = 1). The participants included in the gray matter analysis were thus: 140 participants of which 44 functional monolingual native English-speakers (mean age: 21.0 yr, SD: 2.0, 26 female) who reported limited knowledge of a second language (i.e. complete beginner or elementary status) and 96 functional bilinguals (mean age: 23.7 yr, SD: 3.8, 69 female) who reported to be proficient in 2, but not more, languages. An additional 6 were excluded from the white matter analyses due to data quality issues. The remaining 134 participants for the white matter analyses included 42 native English-speakers (mean age: 20.9 yr, SD: 2.9, 25 female) who were considered functionally monolingual and 92 functional bilinguals (mean age: 23.8 yr, SD: 3.8, 67 female). Note that these distinctions between mono- and bilinguals are for descriptive purposes only. All main analyses were conducted on the combined dataset using continuous measures of language experience (see following section).

Participants provided informed consent prior to participation in the study. All participants were right-handed (as assessed by the Edinburgh Handedness Inventory; Oldfield 1971), had normal or corrected-to-normal vision, no history of head injuries resulting in concussion, and no condition for which neurological damage was a feature, e.g. epilepsy. The study was conducted following the guidelines of the British Psychological Society code of ethics and was approved by the Science, Technology, Engineering, and Mathematics (STEM) Ethical Review Committee for the University of Birmingham.

### Demographic measures and indices of bilingual experience

Participants completed a series of language and socio-demographic background measures. Their language background and daily language use/exposure patterns were measured with the Language and Social Background Questionnaire (LSBQ; Anderson et al. 2018b), a slightly amended version of the Switching Experience and Environments Questionnaire (SEEQ; adapted from Rodríguez-Fornells et al. 2012; Hartanto and Yang 2016), Oxford Quick Placement Test (OQPT; Geranpayeh 2003), the National Adult Reading Test (NART; Nelson and Willison 1991), and a series of verbal fluency tasks in which participants were given 1 min to list as many words as they could according to a certain rule (words beginning with “S” or “F” sound, or words from the semantic categories “animals” or “food”). Bilingual participants performed each task in their first and second language, while monolingual participants performed the tasks in their first language only. Additional measures such as performance in control tasks were taken in the larger project but have been (Carter et al. 2023), or will be, discussed elsewhere.

Responses from these background measures were entered into a confirmatory factor analysis (CFA) which specified the 4 latent variables from the UBET framework (for further details of this analysis, the reader is referred to Carter et al. 2023). These were (i) duration of bilingual language use, (ii) intensity and diversity of language use (hereafter intensity/diversity), (iii) language switching, and (iv) relative language proficiency. The CFA was implemented via the *lavaan* package (Rosseel 2012) in R (version 4,1,2; R Core Team 2021). Scores were derived per participant from each of the calculated factors from the CFA for further analysis, via the LavPredict function within the *lavaan* package. For each factor, a higher score indicates a greater degree of engagement (e.g. longer duration of bilingual experience, more frequent switching, higher intensity of use of both languages, etc.).

As stated above, due to the high overlap in degree of variance explained in modeling between the relative proficiency and intensity/diversity variables (Carter et al. 2023), the relative proficiency factor score was not included in the final statistical analyses.

### MRI data acquisition

MRI data were collected on a 3 T Siemens Prisma scanner with a 32-channel head coil and Syngo software. Participants first underwent a T1-weighted MPRAGE scan (voxel size = 1.0 mm isotropic, TR = 2000 ms, TE = 2.03 ms, flip angle = 8°, FOV = 256 × 256 × 208 mm, acquisition time = 4:54 min). Participants then underwent a diffusion-weighted imaging (DWI) scan (92 slices, voxel size 1.7 mm isotropic, TR = 3500 ms, TE = 86 ms, flip angle 90°, 54 directions, phase encoding A>>P, acquisition time = 3:29 min). For preprocessing purposes, a reverse phase-encoded DWI scan with the otherwise same parameters was run directly after the first DWI scan. The total duration of MR protocol was 12:06 min.

### MRI data preprocessing - cortical gray matter

Cortical GMV was assessed via a VBM analysis, performed using FSL version 6.0.1 (Jenkinson et al. 2012). First, brain images were extracted from T1-weighted images using ANTsPyNet (Tustison et al. 2021). Then the brain-extracted images were segmented into gray matter, white matter, and cerebrospinal fluid via FAST. For better accuracy of registration, a study-specific template was created with equal numbers of monolingual and bilingual participants (44 from each group), randomly chosen from all participants. The gray matter images of the selected participants were affine-registered to the GM CIBM-152 template and then concatenated and averaged to create the template. All native gray matter images were nonlinearly registered to the template and Jacobian modulated. The resulting images were smoothed by full-width half-maximum, with sigma = 3 mm.

Masks were generated for several bilateral language-related regions of interest (ROIs), defined by the latest language network atlas from EvLab (Lipkin et al. 2022). This atlas was derived from functional magnetic resonance imaging (fMRI) data from 806 individuals who underwent a validated language localizer. The resulting atlas allows for the estimation of the probability that any location within a common space belongs to the language network. The ROIs included the IFG, bilateral MFG, bilateral angular gyrus (AG), and bilateral MTG. Masks of the cerebellum and ACC were also extracted. Using these masks, GMV for each ROI was extracted for further statistical analysis.

### Subcortical gray matter

The FSL-Integrated Registration and Segmentation toolbox (FIRST) was used for the segmentation of subcortical structures (Patenaude et al. 2011). The automatically extracted structures included the bilateral hippocampus, amygdala, caudate, putamen, pallidum, nucleus accumbens, and thalamus, which were segmented from the T1-weighted images. The segmented images were manually checked after the automated segmentation. Volumes of each structure (bilaterally) were extracted using the volume extraction pipeline within the FIRST toolbox. Subcortical volumes were corrected for total intracranial/brain volume (TIV/TBV), which was calculated from the extracted total brain volumes from brain extraction (see above section). The corrected subcortical volumes were carried forward for further analyses. Given predictions made by the UBET framework, only the caudate nucleus, putamen, and thalamus were included in the final statistical analyses.

### White matter

Diffusion tensor imaging (DTI) data were processed using standard pipelines in FSL. Data were first preprocessed with the *topup* (Andersson et al. 2003) and *eddy* pipelines (Andersson and Sotiropoulos 2016) to account for susceptibility distortions, eddy current distortions, and any signal outliers. Following preprocessing, a tensor model was fit for each participant using the *dtifit* function within the FMRIB’s Diffusion Toolbox (FDT) pipeline (Behrens et al. 2007). Tract-based spatial statistics (TBSS) analyses were run using the standard pipeline within FSL to generate fractional anisotropy (FA) values for each participant. All subjects were first nonlinearly registered to the MNI standard template. From this, a mean FA image was then rendered and skeletonized. Finally, FA values of all participants were projected onto the skeletonized image. FA values within these tracts were extracted using masks generated from the Juelich histological atlas. The extracted tracts of interest (TOIs) for statistical analysis were the bilateral IFOF, the SLF), and the CC.

### Statistical analyses

As we wished to examine the degree to which the relationships between neuroanatomical outcomes and bilingual experiences were nonlinear, generalized additive models (GAMs) were fitted using the *mgcv* package in R (Wood 2011; Wieling 2018). All models regressed a response variable (i.e., GMV and/or FA values) against the predictor variables of duration, intensity/diversity, language switching, age, and sex (the final 2 being nuisance covariates). The model included smooth terms for all predictor variables. The smooth terms for duration, intensity/diversity, language switching, age, and sex were fitted using a thin plate regression spline s with k knots while sex was fitted using random effect. The basis dimension (k) was also checked in the model and the results showed that k = 10 was appropriate for all predictor variables. The Gaussian error distribution with identity link function was assumed. This led to the following model:

*gam(target ~ s(Duration,k = 10) + s(Intensity_Diversity,k = 10) + s(Language_Switching,k = 10) + s(Age,k = 10) + s(Sex, bs = “re”), data = data).*

## Results

Several significant relationships were found between the bilingual experience factors and neuroanatomical outcomes. All models were properly fitted, as evidenced by appropriate k-index values and an estimated degree of freedom that was smaller than the reference degree of freedom. For sake of brevity, we report only the significant relationships. Table 1 displays for each ROI all significant and nonsignificant predictor variables. Gray matter volume of cortical ROIs was predicted by both intensity/diversity and language switching. FA values in specific tracts were predicted by duration of bilingual experience and intensity/diversity of engagement. Finally, subcortical regions were predicted by language switching and duration of second language usage. We describe these relationships in more detail in what follows.

**Table 1:** Output from all GAM analyses showing significant effects in bold.

**ROI/TOI**	**Measure**	**Smooth terms**	**edf**	**Ref.df**	**F**	***P*-value**
R IFG (ROI5)	GMV	Duration	1.000	1.000	0.068	0.794
		**Intensity/diversity**	**4.736**	**5.795**	**4.091**	**0.001**
		Language switching	1.914	2.425	1.776	0.179
		**Age**	**1.378**	**1.664**	**9.704**	**0.002**
		Sex	0.000	1.000	0.000	0.561
R MFG (ROI6)	GMV	Duration	1.000	1.000	1.391	0.240
		**Intensity/diversity**	**5.046**	**6.167**	**2.728**	**0.016**
		**Language switching**	**5.164**	**6.260**	**2.211**	**0.038**
		Age	1.704	2.125	2.522	0.088
		Sex	0.000	1.000	0.000	0.466
R AG (ROI8)	GMV	Duration	1.000	1.000	1.523	0.219
		**Intensity/diversity**	**1.000**	**1.000**	**5.387**	**0.022**
		Language switching	1.984	2.530	1.721	0.174
		**Age**	**1.000**	**1.000**	**4.403**	**0.038**
		Sex	0.000	1.000	0.000	0.935
L pMTG (ROI3)	GMV	Duration	3.028	3.744	0.660	0.503
		Intensity/diversity	1.000	1.000	2.729	0.101
		**Language switching**	**2.348**	**2.966**	**2.777**	**0.039**
		Age	1.000	1.000	0.076	0.783
		**Sex**	**0.898**	**1.000**	**9.672**	**0.001**
R pMTG (ROI7)	GMV	Duration	1.000	1.000	0.285	0.594
		Intensity/diversity	1.000	1.000	3.892	0.051
		**Language switching**	**2.617**	**3.302**	**3.379**	**0.020**
		Age	3.519	4.377	0.830	0.554
		Sex	0.232	1.000	0.281	0.269
L AG (ROI4)	GMV	Duration	1.000	1.000	1.529	0.218
		Intensity/diversity	1.000	1.000	3.835	0.052
		**Language switching**	**2.536**	**3.210**	**4.066**	**0.007**
		**Age**	**1.000**	**1.000**	**5.130**	**0.025**
		**Sex**	**0.908**	**1.000**	**10.098**	**0.001**
L SLF	FA	**Duration**	**6.329**	**7.454**	**2.511**	**0.015**
		Intensity/diversity	1.000	1.000	0.946	0.333
		Language switching	5.191	6.257	1.403	0.221
		Age	1.000	1.000	3.124	0.080
		Sex	0.000	1.000	0.000	0.779
R SLF	FA	**Duration**	**6.070**	**7.202**	**2.427**	**0.020**
		**Intensity/diversity**	**1.000**	**1.000**	**4.454**	**0.037**
		Language switching	5.722	6.825	1.507	0.169
		**Age**	**1.000**	**1.000**	**4.943**	**0.028**
		Sex	0.000	1.000	0.000	0.462
L Caudate	GMV	**Duration**	**1**	**1**	**4.366**	**0.039**
		Intensity/diversity	3.458	4.24	2.146	0.075
		Language switching	4.016	4.909	1.931	0.096
		**Age**	**1**	**1**	**5.507**	**0.02**
		**Sex**	**1**	**1**	**46.97**	**0.001**

ROI/TOI abbreviations: IFG = inferior frontal gyrus, AG = angular gyrus, MFG = medial frontal gyrus, pMTG = posterior medial temporal gyrus, SLF = superior longitudinal fasciculus.

### Relationship between gray matter volume and bilingual experience variables

**Fig. 2:**
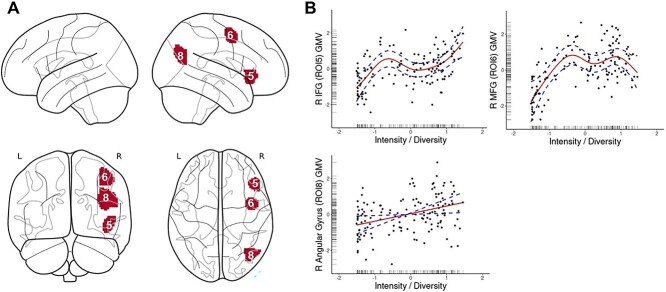
Dynamic relationships between intensity and diversity of engagement in bilingual experience and GMV in cortical ROIs. (A) A render of the ROIs in which GMV was predicted by intensity of bilingual engagement, including right IFG, right mediofrontal gyrus, and right AG. (B) Output of the significant relationships from GAMs. The solid line represents the predicted relationship between diversity/intensity of bilingual engagement and GMV. The dashed lines represent the upper and lower bound of 95% confidence intervals.

Intensity/diversity of bilingual use positively predicted GMV in the right IFG (ROI 5), the right MFG (ROI 6), and the right AG (ROI 8) (Fig. 2).

**Fig. 3:**
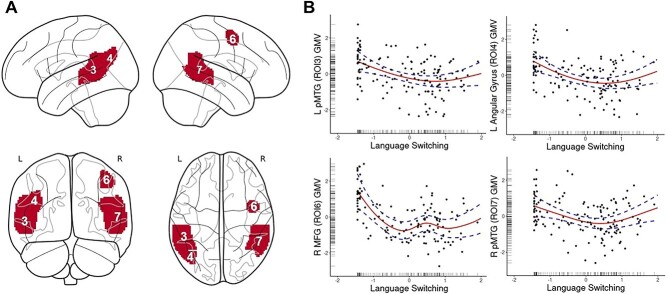
Dynamic relationships between degree of language switching and GMV in cortical ROIs. (A) A render of the ROIs in which GMV was predicted by degree of language switching, including left posterior MTG, right posterior MTG, AG, right MFG. (B) Output of the significant relationships from GAMs. The solid line represents the predicted relationship between the language switching degree and gray matter volume. The dashed lines represent the upper and lower bound of 95% confidence interval.

For language switching, we found a significant association with GMV (Fig. 3) within the left posterior MTG (ROI 3), the right posterior MTG (ROI 7), and the left AG (ROI 4). Finally, there was a significant relationship between language switching and GMV in the right MFG (ROI 6), which also showed a relationship with intensity/diversity, as indicated above.

### Relationship between white matter microstructure and bilingual experience variables

**Fig. 4:**
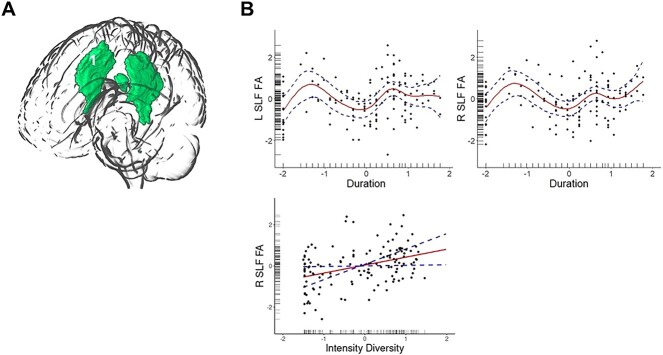
Dynamic relationships between duration of bilingual experience and white matter microstructure (FA values) in TOIs. (A) A render of the SLF in which FA values were predicted by duration of bilingual experience and by intensity/diversity. (B) Output of the significant relationships from GAMs. The solid line represents the predicted relationship between duration and intensity/diversity of bilingual experience and GMV. The dashed lines represent the upper and lower bound of 95% confidence interval.

Within the left SLF, there was a significant association between FA values and duration (Fig. 4). For the right SLF, there were significant associations between FA and duration, intensity/diversity as well as age.

### Relationship between subcortical volumes and bilingual experience variables

**Fig. 5:**
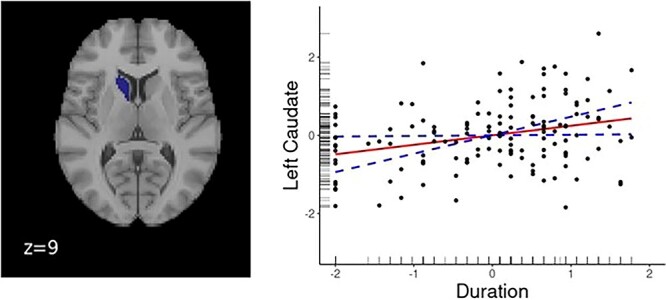
Dynamic relationships between duration of bilingual language use and GMV in the caudate nucleus. (Left) A render of the left caudate. (Right) Output of the significant relationships from GAMs. The solid line represents the predicted relationship between duration of bilingual experience and GMV. The dashed lines represent the upper and lower bound of 95% confidence interval.

Duration of bilingual experience significantly and positively predicted the volume of the left caudate (Fig. 5), as did sex and age.

### Power analysis

**Fig. 6:**
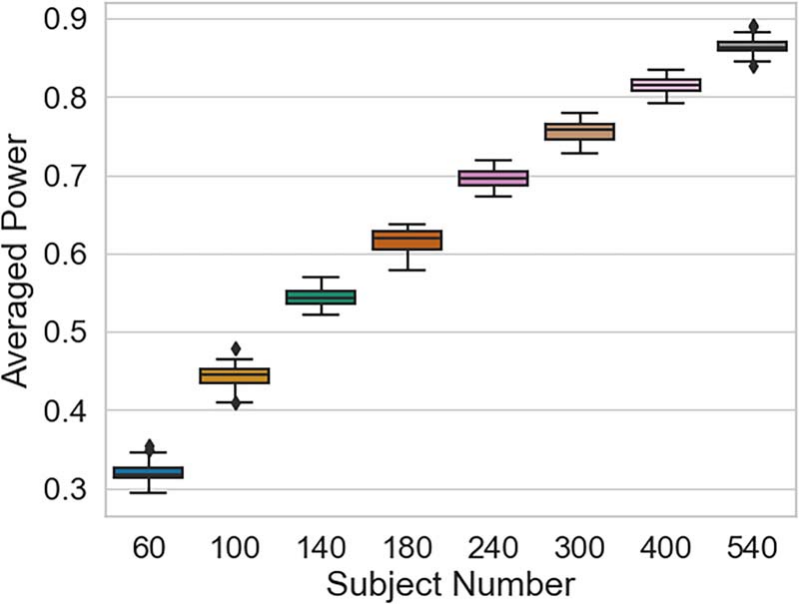
Results of the simulation-based power analysis. The different boxes represent the obtained power for each calculated sample size.

To inform sample size and study design decisions of possible future studies, we used our findings to investigate the power of detecting a non-zero effect using the same factors as those used herein. To this end, we conducted a simulation-based power analysis using the same models as those applied within this study. This analysis showed future studies would need an approximate sample size of 140 participants to yield a power of 0.54 (SD = 0.01), with a power of 0.8 (SD = 0.012) requiring a sample size of ~400 participants (see Supplementary Information for details) (see Fig. 6).

## Discussion

**Fig. 7:**
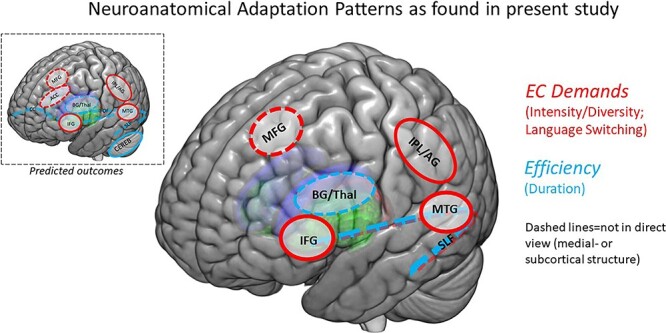
The combined neuroanatomical effects found within the present study, which largely overlap with the predictions of the UBET framework. Red shapes indicate regions implicated in adaptations to increased EC demands, blue lines indicate regions/tracts implicated in adaptations toward efficiency (as predicted by UBET). Picture inset at upper left depicts the original neuroanatomical predictions of UBET. ROI abbreviations: ACC = anterior cingulate cortex, IFG = inferior frontal gyrus, IPL/AG = inferior parietal lobule/angular gyrus, MFG = medial frontal gyrus, MTG = medial temporal gyrus, BG/Thal = basal ganglia (caudate/putamen)/thalamus, Cereb = cerebellum, CC = corpus callosum, IFOF = inferior fronto-occipital fasciculus, SLF = superior longitudinal fasciculus.

The aim of the present study was to directly assess the specific predicted neuroanatomical adaptations to disparate bilingual experiences proposed by the UBET framework. The present results largely support UBET’s predictions (Fig. 7). Specifically, increased *intensity of engagement* and frequency of *controlled language switching* both correlated with adaptations toward more effectively handling increased *EC demands.* More specifically, this manifested with increases in GMV in relevant cortical regions. Prolonged *duration of bilingual experience* was associated with neuroanatomical adaptations toward *efficiency*, specifically adaptations in subcortical gray matter and white matter microstructure in tracts connecting frontal and posterior regions, bilaterally. In what follows, we discuss the present findings and the larger implications for the nature of bilingualism-induced neural plasticity.

### Intensity and diversity of bilingual experience and language switching are reflected in adaptations to increased control demands

UBET predicts adaptations in GMV across several cortical regions in response to increased EC demands (in red in Fig. 1). Both increased intensity of engagement with bilingual experience and more frequent controlled language switching are proposed to contribute to these EC demands, and thus would predict increases in gray matter in cortical regions implicated in the relevant processes. The results found in the present study support those predictions, particularly for intensity/diversity of bilingual engagement.

The IFG and AG/IPL are both heavily implicated in language selection and control processes and thus EC demands (Calabria et al. 2018). Both have also been associated with increases following acquisition and use of an additional language (e.g. Legault et al. 2019). Similarly, the medial frontal cortex is involved in a range of EC processes, particularly adaptations in goal-directed behavior (Ridderinkhof et al. 2004). The positive association with intensity/diversity of language engagement and gray matter volumetric patterns in these regions supports the predictions of UBET (as well as the ACH and DRM) of cortical adaptations to handle the associated increased EC demands. The adaptations in the right hemisphere seen for intensity/diversity also support the notion of increased recruitment of the structures in the homologous hemisphere to assist in the processing of the increased cognitive demands associated with bilingual engagement (see e.g. Sabourin 2014).

The structural plasticity effects seen within the bilateral MTG, left AG, and right medial fontal gyrus are dynamic, following a u-shape curve of GMV with increased rate of controlled language switching. That is, volumes of these cortical regions appear to decrease in gray matter with lower degrees of language switching, and then increase at higher rates of language switching. It should be noted that the factor score for language switching predominantly comprised variables measuring controlled language switching (Carter et al. 2023). Higher scores within this factor, thus, largely reflect increased EC demands, as opposed to a dense code-switching context where EC demands are predicted to decrease (Green and Abutalebi 2013). The dynamic relationship between language switching and GMV reflects an adaptation within the EC network to optimize the accommodation of the changing/increasing EC demands associated with changes in language switching experience. This, then, provides at least partial support for the predictions of the UBET framework for neuroanatomical adaptations to language switching (DeLuca et al. 2020). This result also overlaps with predictions of the DRM, specifically increased/novel control demands corresponding to gray matter adaptations in cortical regions (Pliatsikas 2020).

The positive relationship between intensity of bilingual engagement and FA values in the right SLF may seem counterintuitive at first, given that white matter plasticity is argued to reflect efficiency of processing. However, this may also reflect a transition toward increased efficiency of processing. The UBET framework does predict situations whereby increased intensity of engagement may shorten the latency by which adaptations toward increased efficiency might occur. We also cannot preclude the possibility of adaptations toward efficiency and EC demands to overlap in latency, particularly as it pertains to macroscale adaptations in brain structure. Given the present pattern of results, it is possible that the efficiency of communication between regions (and adaptations in white matter microstructure) may occur before cortical regions would return to “baseline” (Lövdén et al. 2013; see also the prediction of the DRM in Pliatsikas 2020). As the SLF connects a host of cortical regions (Nakajima et al. 2020), including the 3 which were predicted to be modulated by intensity of bilingual experience (AG, MFG, and IFG), this effect supports this notion. However, more data, particularly from longitudinal studies are required to further assess how these latencies play out through time and with shifts in degree of engagement.

Taken together, the neuroanatomical adaptation patterns for both language switching and intensity and diversity of language use reflect adaptations toward more effectively handling the dynamic EC demands associated with these bilingual experiences, in line with predictions of UBET.

### Duration of bilingual experience increases efficiency in EC

Recall that UBET proposes that adaptations toward increased efficiency in EC would involve greater structural plasticity in white matter microstructure and subcortical gray matter (in blue in Figs 1 and 6). Furthermore, prolonged duration of bilingual experience would relate to such a pattern of efficiency-based adaptations. Specifically, reliance within the EC network is proposed to shift away from cortical structures and toward subcortical structures (more automated and efficient processing, see also BAPSS and DRM for similar predictions). Furthermore, plasticity is proposed to occur within the white matter tracts that connect the regions involved in EC (as communication between them becomes more efficient). The pattern of neuroanatomical results predicted by duration of bilingual experience supports this proposed relationship.

The increase and subsequent stabilization of FA values in the bilateral SLF with prolonged duration of bilingual experience can be interpreted as a shift in reliance toward increased efficiency in handling the EC demands associated with bilingual experience. The SLF connects frontal, parietal, and posterior regions in the brain, many of which have been implicated in EC processes (Nakajima et al. 2020). The changes in FA values in this tract indicate a restructuring of white matter microstructure, facilitating increased efficiency of communication between the regions this tract connects. The present results also overlap with previous work showing general effects of bilingualism and increased L2 proficiency in this tract (Pliatsikas et al. 2015; Kuhl et al. 2016; Singh et al. 2018). Moreover, structural resilience against aging has been observed as an effect of bilingual experience in the SLF (Luk et al. 2011; Anderson et al. 2018a; DeLuca and Voits 2022). The present results also support the predictions of the UBET and DRM, both of which propose adaptations in white matter microstructure as a marker of increasing efficiency, commensurate with prolonged duration of engagement with multiple languages.

The positive correlation seen between volume in the left caudate with duration of bilingual language use also supports both UBET and predictions of several models within the field of bilingualism and neurocognition. The caudate is argued to be a hub within the EC network and has been implicated in both language- and domain-general EC processes (Stocco et al. 2014; Li et al. 2015; Calabria et al. 2018). Furthermore, prolonged duration of bilingual experience is predicted to shift reliance away from cortical regions and toward the basal ganglia with increased automation and efficiency of handling demands, in line with UBET, BAPSS, CRM, and DRM (Stocco et al. 2014; Grundy et al. 2017; DeLuca et al. 2020; Pliatsikas 2020). This shift in reliance would manifest (at least initially in this process of transition) in greater GMV in this structure, which the present data support. The effect in the caudate also overlaps with other studies showing effects of bilingual experience in this structure (Pliatsikas et al. 2017; DeLuca et al. 2019a; Korenar et al. 2023).

### Future directions and conclusions

Although many of the regions implicated in UBET’s predictions were found to be affected in the present study, some notable exceptions exist where effects of bilingual experience were not found, including the CC, ACC, and cerebellum. It is worth revisiting the language demographics of the cohort tested in our study, which have implications for the nature and range of the language exposure patterns within this sample and thus the associated EC demands and neuroanatomical adaptations. Recall that our cohort included native English-speakers with little to no exposure to additional languages (i.e. functional monolinguals) and those who spoke English and an additional language, all living in a largely English-dominant environment at time of testing. As discussed in recent models like the Systems Framework of Bilingualism (Titone and Tiv 2023), societal language patterns and specific local patterns of language exposure and use will likely delimit the range of possible (bilingual) experiences which can be captured within any given cohort or group. The neuroanatomical adaptation patterns to the further end of the spectrum of such experiences (e.g. intensity of use, switching patterns) would thus likely not have been captured within the present study. Taking the present study cohort as an example, inclusion of participants with longer duration of (and more intensive) exposure to multiple languages might have provided sufficient variability across the cohort to observe structural effects within the cerebellum, more white matter tracts, and/or subcortical structures (Grundy et al. 2017; DeLuca et al. 2020; Pliatsikas 2020). Further tests of the UBET framework would thus ideally include populations with a more diverse range of language experiences (e.g. more variability in degree of language switching, availability of languages across social/professional settings) to capture these potential effects.

The aim of this study was to directly test, for the first time, the neuroanatomical predictions of the UBET framework. Neuroanatomical patterns predicted by intensity and diversity of bilingual experience are indicative of adaptations toward handling increasing EC demands. Distinctly, the neuroanatomical patterns predicted by duration of bilingual experience are indicative of adaptations toward increased efficiency of handling these demands. Taken together, the results support the predictions of UBET. Moreover, the data speak to growing calls for theory-driven research in future work on bilingualism-induced neurocognitive outcomes. A growing body of research has shown that distinct language experiences have implications for the nature and degree of adaptations to them. While this direction is promising, these results and future research need to be couched in and guided by solid theoretical grounding to further advance our understanding. This study presents a method by which we can more rigorously test the predictions for individual differences in bilingualism-induced neuroanatomical adaptations and represents a step forward in terms of how we can understand neural and cognitive implications of specific bilingual experiences.

## Supplementary Material

BrainstrucUBET_supplementary_info_bhae029

## Data Availability

Raw data and analysis code for this study are available on the Open Science Framework Server (https://osf.io/ab8u3/).
