# Supplementary material for: Mapping individual aspects of bilingual experience to adaptations in brain structure
Source: Cereb Cortex. 2024 Feb 13;34(2):bhae029. doi: 10.1093/cercor/bhae029 (PMC10872681; doi:10.1093/cercor/bhae029)
Supplement: BrainstrucUBET_supplementary_info_bhae029 [file brainstrucubet_supplementary_info_bhae029.docx]

**Supplementary materials**

We conducted a simulation-based power analysis to inform future study design. First, we fit separate statistical models for monolingual and bilingual participants using the current dataset to generate the simulated dataset. The simulated dataset contains half monolinguals and half bilinguals. We then applied the same generalized additive models (GAMs) to the simulated datasets. To calculate power, we recorded the percentage of times the null hypothesis of zero mean was successfully rejected out of 100 simulated analyses. We then averaged the powers across factors, such as language switching, intensity diversity, and duration, for a simplified presentation.

To examine how power varies with the number of subjects, we performed the above process with different sample sizes. The entire simulation was run 25 times to obtain a distribution of power values. Based on the simulation study, we assessed the Type II error rate and provided valuable insights for determining appropriate sample sizes in future studies.

*Results:*


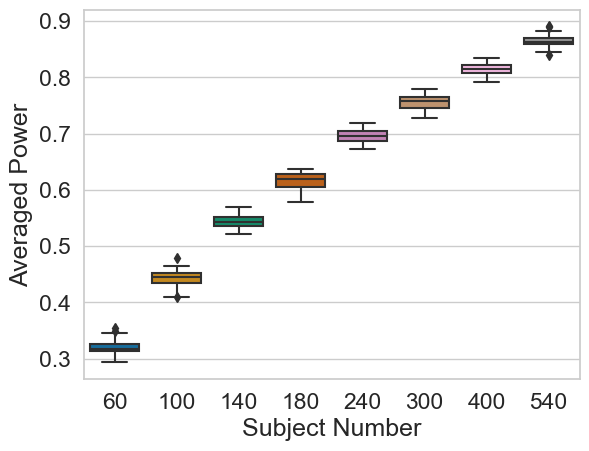
We investigated the power of detecting the non-zero effect for all included factors to inform the Type-II error rate for possible future studies using simulation-based power analysis. We simulated data sets with different numbers of participants using R. The power is calculated by the percentage of those simulated data sets which rejected the null hypothesis. The figure below shows the obtained power of included factors as a function of the number of participants included. When the subject number is 140, the respective power is 0.54 (SD=0.01).
